# Polyphenol-Loaded Nanodevices as Innovative Therapeutic Strategies for Dry Eye Disease: Advances and Perspectives

**DOI:** 10.3390/antiox14111280

**Published:** 2025-10-25

**Authors:** Raffaele Conte, Ilenia De Luca, Anna Calarco, Mauro Finicelli, Gianfranco Peluso

**Affiliations:** 1 Research Institute on Terrestrial Ecosystems (IRET)-CNR, Via Pietro Castellino 111, 80131 Naples, Italy; raffaele-conte@cnr.it (R.C.); gianfranco.peluso@unicamillus.org (G.P.); 2 National Biodiversity Future Center (NBFC), 90133 Palermo, Italy; 3 IRCCS SYNLAB SDN, Via G. Ferraris, 144, 80146 Naples, Italy; ilenia.deluca@synlab.it; 4 Faculty of Medicine and Surgery, Saint Camillus International University of Health Sciences, Via di Sant’Alessandro 8, 00131 Rome, Italy

**Keywords:** dry eye disease, polyphenols, nanotechnology, oxidative stress, anti-inflammatory therapy

## Abstract

**Background:** Dry Eye Disease (DED) is a multifactorial ocular disorder characterized by tear film instability, inflammation, oxidative stress, and ocular surface damage. Current therapeutic options often provide only temporary relief and are limited by poor patient compliance and inadequate drug retention at the ocular surface. **Aim:** This review aims to critically analyze the therapeutic potential of polyphenols and their nanoencapsulated formulations for the management of DED, focusing on pharmacological mechanisms, formulation strategies, and translational implications. **Methods:** A comprehensive literature search was conducted in PubMed, Scopus, and Web of Science databases using combinations of the following keywords: “dry eye disease,” “polyphenols,” “antioxidants,” “nanocarriers,” “ocular delivery,” and “bioavailability.” Studies published in English from 2000 to 2024 were considered. Inclusion criteria encompassed experimental, preclinical, and clinical studies evaluating polyphenol-based formulations for ocular application, while reviews without original data or studies unrelated to DED were excluded. **Results:** The analysis identified EGCG, curcumin, resveratrol, and quercetin as the most extensively investigated polyphenols, exhibiting antioxidant, anti-inflammatory, and cytoprotective activities through modulation of cytokines, reactive oxygen species, and immune signaling pathways. Nanoformulations such as lipid nanoparticles, micelles, and cyclodextrin complexes improved solubility, stability, ocular retention, and bioavailability, leading to enhanced therapeutic efficacy in preclinical DED models. **Conclusions and Future Perspectives:** Polyphenol-loaded nanocarriers represent a promising approach for improving the management of DED by enhancing local drug delivery and sustained release. However, further clinical studies are needed to assess long-term safety, scalability, and regulatory feasibility. Future research should focus on optimizing formulation reproducibility and exploring personalized nanotherapeutic strategies to overcome interindividual variability in treatment response.

## 1. Introduction

Dry Eye Disease (DED) is a chronic and multifactorial disorder of the ocular surface and tear film, which leads to symptoms of discomfort, visual disturbance, and tear film instability with potential damage to the ocular surface [1]. It is often associated with increased osmolarity of the tear film and inflammation of the ocular surface, creating a self-perpetuating “vicious circle” of damage [2]. Epidemiological studies estimate that DED affects between 5% and 50% of the global population, depending on diagnostic criteria, age, sex, and geographic region, with a higher prevalence among elderly individuals and postmenopausal women. The condition exerts a substantial socioeconomic burden, contributing to reduced visual performance, work productivity, and quality of life [1]. This condition affects millions of individuals worldwide and significantly impacts quality of life and productivity. Conventional therapies for DED—including artificial tears, corticosteroids and cyclosporine—often provide only symptomatic relief and may present limitations in terms of efficacy, safety, or patient adherence [3]. While artificial tears restore surface moisture, they do not address the underlying inflammatory and oxidative mechanisms. Immunomodulators such as cyclosporine A or lifitegrast can reduce inflammation but are often associated with delayed onset of action and ocular irritation, highlighting the need for novel treatments that can simultaneously target multiple pathogenic pathways [1,3]. In recent years, increasing attention has been paid to the role of oxidative stress, mitochondrial dysfunction, and immune dysregulation in DED pathogenesis, opening the door to novel therapeutic approaches targeting these mechanisms, such as those offered by natural bioactive compounds with multi-targeted actions [4]. In particular, the imbalance between reactive oxygen species (ROS) production and antioxidant defense systems has been implicated in tear film instability, epithelial apoptosis, and chronic inflammation. Pro-inflammatory cytokines such as IL-1β, IL-6, TNF-α, and matrix metalloproteinases (MMPs) contribute to epithelial barrier disruption and neurosensory abnormalities, further aggravating disease progression [1,4]. Polyphenols, a diverse class of bioactive compounds found in fruits, vegetables, and medicinal plants, possess well-documented anti-inflammatory, antioxidant, immunomodulatory, and anti-apoptotic properties [5]. These activities make them promising candidates for the treatment of inflammatory and degenerative ocular diseases, including DED. Among them, compounds such as epigallocatechin gallate (EGCG), curcumin, resveratrol, and quercetin have demonstrated the ability to modulate NF-κB and Nrf2 signaling pathways, reduce oxidative stress, and suppress pro-inflammatory mediators in ocular cell models and animal studies [5]. However, the therapeutic application of polyphenols in ophthalmology is hindered by their limited aqueous solubility, poor chemical stability, and low ocular bioavailability [6]. In addition, their pharmacokinetic behavior is affected by rapid metabolism, poor corneal permeability, and tear fluid washout, which limit the concentration of active compounds at the ocular surface [6]. To address these pharmacokinetic limitations, several nanotechnology-based strategies have been explored. Nanocarriers such as solid lipid nanoparticles (SLNs), nanostructured lipid carriers (NLCs), micelles, and polymeric or cyclodextrin-based systems have been developed to improve polyphenol delivery, retention time, and bioactivity on the ocular surface [7,8,9]. These systems can enhance solubility, protect polyphenols from degradation, and provide sustained release profiles, thereby improving patient adherence and therapeutic efficacy in chronic ocular disorders such as DED. In this review, we focus on four representative polyphenols—epigallocatechin gallate (EGCG), curcumin, resveratrol, and quercetin—that have been investigated in preclinical studies for the treatment of DED using nanotechnological delivery systems. Their molecular effects on tear film regulation, inflammation, and oxidative damage are examined, along with formulation strategies and current limitations for translational development. To the best of our knowledge, this is the first review to systematically compare nanoformulated polyphenols specifically for DED and critically evaluate the formulation–efficacy relationship.

## 2. Methods

This narrative review was conducted in three main stages: literature search, study selection, and synthesis of relevant findings. A comprehensive search was performed using the PubMed, Scopus, EMBASE, Web of Science, ScienceDirect, and Google Scholar databases to identify studies related to Dry Eye Disease (DED), polyphenols, and nanocarrier-based delivery systems. The final search was completed in August 2025 and included peer-reviewed articles, reviews, reports, and electronic books published in English. The following keywords and their combinations were used: “dry eye disease,” “ocular surface,” “polyphenols,” “antioxidants,” “nanocarriers,” “nanoformulations,” “ocular drug delivery,” “bioavailability,” and “oxidative stress.” After removing duplicates, the remaining abstracts were screened for relevance to the topic and compliance with the inclusion criteria following the PRISMA (Preferred Reporting Items for Systematic Reviews and Meta-Analyses) guidelines to ensure transparency and reproducibility of the search process. Eligible studies were those investigating polyphenol-based formulations or nanotechnological delivery systems with relevance to DED pathophysiology, therapeutic mechanisms, or ocular drug delivery. Studies not focused on DED or lacking data on polyphenols or nanocarriers were excluded. The selected publications were reviewed in full, summarized, and synthesized to provide an integrated and critical overview of the current evidence. In addition, a bibliographic network analysis was performed using VOSviewer software (version 1.6.20) to map the intellectual structure of the reviewed literature. This analysis allowed identification of major research clusters, keyword co-occurrences, and citation networks, revealing trends and gaps in the field. The bibliographic network highlighted the prominence of terms such as oxidative stress, nanocarriers, bioavailability, and specific polyphenols (EGCG, curcumin, resveratrol, quercetin) in DED-related research. It also revealed that studies on polymeric nanoparticles and lipid-based carriers dominate the field, while certain nanoformulation types (e.g., inorganic nanoparticles) are less explored, suggesting opportunities for future research.

Given the narrative nature of this review, formal registration or documentation of the search strategy in a systematic review database was not required.

## 3. Dry Eye Disease (DED)

**Figure 1:**
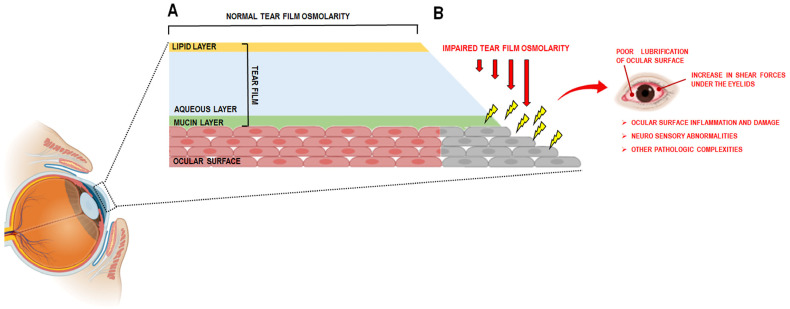
(**A**) schematic representation of the normal tear film; (**B**) DED is characterized by the loss of the tear film (TF), which results in an impaired tear osmolarity and a poor lubrification of ocular surface with a concomitant increase in shear forces under the eyelids. This leads to ocular surface inflammation and damage, neuro sensory abnormalities and other pathologic complexities. This image was created with BioRender.com (accessed on 23 May 2025).

Dry Eye Disease (DED), also termed dry eye syndrome, keratoconjunctivitis sicca, or chronic inflammatory ocular surface disease, represents a significant global ocular health challenge. DED is a multifactorial disorder primarily affecting the ocular surface. The most widely accepted definition was established by the Tear Film and Ocular Surface Society International Dry Eye Workshop II (TFOS DEWS II, 2017), which characterizes DED as a loss of tear film (TF) homeostasis, resulting in tear hyperosmolarity, decreased ocular surface lubrication, and increased mechanical shear forces under the eyelids [10] (Figure 1). These alterations induce ocular surface inflammation, epithelial damage, neurosensory abnormalities, and subsequent pathological sequelae [11]. Clinically, DED manifests with symptoms including ocular discomfort, redness, burning, stinging, dryness, photophobia, foreign body sensation, grittiness, and visual disturbances. It is a leading cause of ophthalmic consultations and significantly impairs patients’ quality of life [12,13]. The prevalence of DED varies between 5% and 50%, depending on diagnostic criteria and studied populations, with an estimated 344 million affected individuals worldwide [14]. Aging is a predominant risk factor, with a noted higher incidence of signs compared to symptoms across decades [15]. Women exhibit a higher susceptibility, with prevalence increasing earlier and more prominently than in men [16,17]. Additional risk factors include systemic comorbidities such as diabetes and autoimmune disorders, exposure to air pollution, and pharmacotherapies for psychiatric conditions [18,19,20,21]. The extensive use of visual display terminals has also been implicated, a concern amplified by increased screen time during the COVID-19 pandemic [12]. Furthermore, improper face mask usage may exacerbate tear evaporation, simulating conditions seen with continuous positive airway pressure therapy [22]. Collectively, these data underscore the pervasive and multifactorial nature of DED as a global public health issue [12].

### 3.1. The Tear Film and the Etiopathogenesis of DED

The tear film (TF) is a specialized, thin fluid layer ranging from approximately 3 to 8 µm in thickness and 3 µL in volume, covering the corneal and conjunctival surfaces. It functions to protect the ocular surface from environmental insults, facilitate eyelid-globe lubrication, and maintain optical clarity [11,12]. The TF is composed of three layers: an outer lipid layer, a middle aqueous layer, and an inner mucinous layer [11,23]. The lipid layer (~10–50 nm thickness) is secreted by the meibomian glands (MBGs), sebaceous glands located in the tarsal plates of the eyelids. This lipid layer consists of a bilayer structure, with non-polar lipids (wax esters, cholesterol esters, steroid esters) interfacing with the air, and polar lipids (O-acyl-ω-hydroxy fatty acids, phospholipids) adjoining the aqueous layer. This lipid film retards aqueous evaporation and preserves tear film stability through modulation of surface tension and viscoelastic properties [24,25]. The aqueous layer, constituting the bulk of the TF, originates from secretions of the main and accessory lacrimal glands, located in the orbital lacrimal fossa. This layer contains water, electrolytes, proteins including immunoglobulins, cytokines, and growth factors, and is supplemented by conjunctival epithelial secretions [11]. It provides essential nutrients, oxygen, and waste removal for the ocular surface, regulates tear osmolarity, and participates in cellular signaling and surface repair mechanisms [24]. The innermost mucin layer comprises glycosylated mucins predominantly secreted by conjunctival goblet cells, with additional transmembrane mucins produced by corneal and conjunctival epithelia. These mucins confer gel-forming and lubricative properties essential to reduce friction and stabilize the TF [11,26]. Notable ocular mucins include MUC1, MUC2, MUC4, MUC5AC, MUC7, MUC13, MUC15, MUC16, and MUC17. TF turnover is approximately 1.7 µL/min, with a 25% renewal rate per minute, influenced by environmental factors such as temperature, humidity, and emotional stimuli [27]. The integrity of the tear film is central to ocular surface health, as it provides lubrication, nourishment, and protection against environmental stressors [12,28]. Disruption of this delicate multilayered structure can lead to two overlapping DED subtypes: aqueous-deficient dry eye (ADDE) and evaporative dry eye (EDE). ADDE is characterized by reduced aqueous tear production due to lacrimal gland dysfunction, including Sjögren’s syndrome (SS)—an autoimmune condition—and non-Sjögren’s etiologies such as lacrimal gland obstruction [10]. Differently, EDE results primarily from increased tear evaporation, often due to meibomian gland dysfunction, lid abnormalities (e.g., blink or aperture dysfunction), allergic conjunctivitis, or vitamin A deficiency (xerophthalmia) [11]. These subtypes frequently coexist and may potentiate each other; for instance, EDE-induced corneal hypoesthesia may reduce reflex lacrimal secretion, worsening aqueous deficiency. Conversely, aqueous deficiency may destabilize lipid spreading, exacerbating evaporative loss [28]. Although useful in early disease characterization, these distinctions blur as DED progresses into a common final pathway marked by tear hyperosmolarity, instability, and chronic inflammation, constituting a self-perpetuating vicious cycle [28].

### 3.2. DED and Inflammation: A Vicious Circle

**Figure 2:**
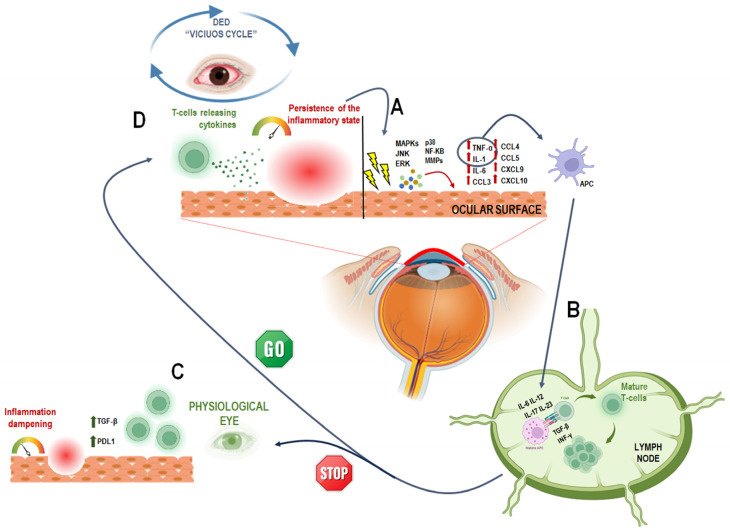
A schematic representation of the DED “vicious circle” triggered by inflammation. (**A**) Irritation caused by environmental or internal factors leads to the release of pro-inflammatory molecules, which activates pro-inflammatory factors, such as Mitogen activated protein kinases (MAPKs), c-Jun N-terminal kinase (JNK), extracellular signal–related kinase (ERK) and p38. These stimulate NF-κB and MMPs, leading to the release of inflammatory cytokines (e.g., TNF-α, IL-1, IL-6) and chemokines (e.g., CCL3, CCL4, CCL5, CXCL9, CXCL10) by the corneal and conjunctival epithelium [18,30,31]. This activates the innate immune response; TNF-α and IL-1 promote the maturation of antigen-presenting cells (APCs). (**B**) mature APCs (mAPCs) migrate to lymph nodes, where they stimulate naïve T cells (TH0) [32,36]. to differentiate into active T cell subtypes (e.g., TH1, TH17) through cytokines like IL-6, IL-12, IL-17, IL-23, TGF-β, and IFN-γ [37,38]. (**C**) These T cells then migrate to the ocular surface, sustaining the inflammation. Normally, regulatory factors like TGF-β and PDL1 help control this process [18,39]. but (**D**) in DED, this regulation fails. As a result, pro-inflammatory T cells maintain inflammation, leading to tissue damage and tear film dysfunction—reinforcing the “vicious cycle” [29]. This image was created with BioRender.com (accessed on 23 May 2025).

Tear hyperosmolarity is the central pathogenic event in DED, directly injuring the ocular surface and initiating inflammatory cascades. The “vicious circle” model describes how TF alterations lead to frictional trauma and activation of innate immune responses, which, if persistent, stimulate adaptive immunity and chronic inflammation [28,29] (Figure 2). Initial insults activate mitogen-activated protein kinases (MAPKs), including JNK, ERK, and p38, which induce transcription factors such as NF-kB and upregulate matrix metalloproteinases (MMPs) and pro-inflammatory cytokines (TNF-α, IL-1, IL-6), chemokines (CCL3, CCL4, CCL5, CXCL9, CXCL10), and T-cell attracting factors [18,30,31]. These mediators promote maturation of antigen-presenting cells (APCs) that migrate to lymph nodes via CCR7 chemotaxis, bridging innate and adaptive immunity by driving naïve T cell differentiation into effector subsets (TH1, TH2, TH17, Treg) [32]. In DED, regulatory mechanisms such as TGF-β secretion by goblet cells and PD-L1-mediated T cell suppression fail, allowing chronic immune activation. TH1 cells produce IFN-γ, inducing goblet cell loss, epithelial apoptosis, and conjunctival hyperplasia, while TH17 cells secrete IL-17, amplifying inflammation, MMP production, and corneal barrier disruption [33,34]. The ensuing inflammatory cascade perpetuates ocular surface damage and tear dysfunction, sustaining the vicious circle [18,29]. Systemic inflammatory diseases such as Sjögren’s syndrome and graft-versus-host disease exacerbate DED by lymphocytic infiltration and fibrosis of lacrimal glands and meibomian glands, further diminishing tear secretion and fostering ocular surface inflammation [35].

### 3.3. DED, Oxidative Stress and Environmental Cues

Oxidative stress plays a synergistic and amplifying role in DED pathogenesis. It arises from an imbalance between reactive oxygen species (ROS) and antioxidant defenses and can be triggered by intrinsic aging processes, mitochondrial dysfunction, and external insults such as ultraviolet light, tobacco smoke, and environmental pollutants. Excess ROS—such as superoxide anion, hydrogen peroxide, and hydroxyl radicals—cause lipid peroxidation, DNA damage, and protein denaturation, which collectively impair ocular surface integrity and lacrimal gland function. This exacerbates TF instability by reducing aqueous production, disrupting mucin expression, and accelerating tear evaporation. Oxidative stress not only damages the epithelial barrier but also amplifies immune activation by enhancing the expression of cytokines and MMPs, further intensifying tissue injury). As a multifactorial condition, DED involves ROS at several etiopathogenic stages: ROS accumulation can activate inflammatory signaling, immune cell infiltration, and may damage the lipid layer of TF or the myelin sheath of ocular surface nerves [40]. Animal models have been instrumental in elucidating these mechanisms. Sod1 knockout (KO) mice, lacking the primary isoform of superoxide dismutase (accounting for 90% of total SOD activity), exhibit lacrimal gland atrophy, fibrosis, immune cell infiltration, increased DNA/lipid oxidation, apoptosis, and mitochondrial dysfunction—all leading to TF alterations and reduced protein secretion [41]. In aged Sod1−/− mice, conjunctival squamous metaplasia and downregulation of mucin genes (Muc1, Muc5a) have also been reported [42]. Meibomian gland dysfunction (MGD) in these mice correlates with increased IL-6, TNF-α, and oxidative markers, highlighting the role of oxidative stress in MGD-associated DED [43]. Similarly, Tet-mev-1 mice overexpressing a mitochondrial ROS-inducing gene exhibit ocular surface damage and impaired aqueous secretion, implicating mitochondrial dysfunction in lacrimal insufficiency [40]. In rat models, prolonged low-humidity exposure increased oxidative markers (8-oxodG, 4-HNE) and fluorescein staining, a clinical marker of DED [44]. Aged rats also show impaired expression of Rab3d, Rab27b, and syntaxin in lacrimal glands, suggesting age-linked oxidative decline affects tear secretion [45]. In humans, oxidative stress in DED has been notably documented in SS, with decreased antioxidant enzymes (SOD, catalase, glutathione peroxidase) correlating inversely with disease severity [46]. Elevated lipid peroxidation products in TF and conjunctiva of SS patients, such as HEL and 4-HNE, further associate with staining scores and inflammatory cell densities, reinforcing the oxidative-inflammation nexus [47]. This dual contribution—mechanical damage from tear hyperosmolarity and biochemical damage from ROS—creates a feedforward loop of inflammation and surface degradation. The result is chronic, self-sustaining disease progression.

Environmental pollutants are critical external sources of oxidative stress. The ocular surface, directly exposed to airborne pollutants, is affected by industrial emissions and fossil fuel combustion byproducts [48]. Epidemiological studies support a strong link between air pollution and DED [49]. An interesting study on OM exposure in human corneal epithelial (HCE) cells and in mouse ocular surface was carried out. In vitro, particulate matter (PM) exposure reduces HCE cell viability, disrupts mitochondrial membrane potential, and increases ROS levels. In vivo, mice exposed to PM develop corneal epithelial damage, goblet cell loss, inflammation, and mucin production deficits via necroptosis [50,51]. Prolonged PM exposure in C57BL/6 mice impairs tear secretion, reduces TF stability, and promotes apoptosis and inflammation in ocular tissues in a dose- and time-dependent manner [52]. Epidemiological data confirm these findings: increased PM2.5, PM10, SO_2_, NO_2_, and CO levels correlate with outpatient visits for DED and symptom aggravation [53,54,55,56]. Beyond pollutants, environmental factors like wind, humidity, and temperature also disrupt TF stability and promote hypoxic and inflammatory responses [19]. In recent years, the contribution of the blue light has attracted increasing attention as an environmental factor potentially involved in pathogenesis of DED. The widespread use of digital screens and LED raised the exposure of these high-energy short waves that can penetrate the cornea and lens reaching the retina, contributing to photochemical damages [57]. The cornea is the first point of contact for the lights entering the eye; thus, blue light can directly act on corneal epithelial cells. This leads to increased ROS levels and IL-1β secretion, which contribute to cellular oxidative damage and the inflammatory response [58]. Moreover, blue light can also reach the retina epithelial cells and contribute to DED by inducing oxidative stress and inflammation. The accumulation of lipofuscin, a by-product of the visual cycle, in retinal pigment epithelial (RPE) cells generates large amounts of ROS under blue light irradiation [59].

Despite its complex pathophysiology, current treatments primarily offer symptomatic relief. Artificial tears aim to restore tear volume but do not interrupt the inflammatory or oxidative cascades. More advanced interventions, as recommended by the TFOS-DEWS II guidelines, include immuno-modulatory agents (e.g., cyclosporine A, lifitegrast), punctal plugs, and tear stimulants to modulate the immune response and restore tear homeostasis. In severe or refractory cases, surgical options such as tarsorrhaphy or salivary gland transplantation may be necessary. The treatment of these phenomena requires tailored approaches aiming at reducing the rates of oxidative damage and inflammation [60,61].

## 4. Conventional Approaches to Treat DED

The therapeutic approach to DED requires a personalized and multimodal strategy aimed at restoring tear film homeostasis, reducing ocular surface inflammation, and improving patient-reported symptoms [62]. Given the multifactorial nature of DED, treatment plans should be tailored to the underlying pathophysiological mechanisms—aqueous deficiency, evaporative loss, or mixed forms—while also considering the severity and chronicity of the disease [62]. Management typically progresses from supportive interventions in mild cases to pharmacological and procedural therapies in moderate-to-severe presentations.

### 4.1. Supportive and Lubricating Therapies

Artificial tears remain the cornerstone of DED management, especially in mild to moderate cases. These topical formulations serve to rehydrate the ocular surface, restore tear film volume, reduce friction, and dilute proinflammatory mediators [63]. Their composition has evolved from simple saline-based solutions to complex formulations mimicking natural tears in terms of osmolarity, viscosity, and lipid content [63]. Hyaluronic acid, due to its viscoelastic properties and epithelial trophic effects, is commonly used to enhance hydration and promote corneal healing [64]. Carboxymethylcellulose and hydroxypropyl methylcellulose act as mucoadhesive polymers, increasing tear film retention time [65]. In evaporative forms of DED, lipid-based artificial tears are employed to stabilize the tear film lipid layer and reduce tear evaporation. These include emulsions containing mineral oils or phospholipids, which aim to replenish the dysfunctional meibomian gland lipid secretion.

### 4.2. Topical Nutraceuticals and Antioxidants

In parallel, growing interest has been directed toward the use of topical nutraceuticals and antioxidant agents [66]. Vitamin A plays a critical role in maintaining the differentiation and integrity of conjunctival and corneal epithelium, and its deficiency is associated with goblet cell loss and keratinization. Topical formulations containing vitamin A have demonstrated improvements in tear breakup time (TBUT) and ocular surface staining [67]. Coenzyme Q10 (CoQ10), a mitochondrial cofactor with antioxidant and antiapoptotic properties, has shown promise in promoting epithelial repair and reducing oxidative stress, particularly in patients with severe DED or associated neurotrophic keratopathy [68]. Trehalose, a disaccharide with osmoprotective and antioxidative activity, acts by stabilizing cell membranes and inhibiting inflammatory pathways, thereby preserving epithelial integrity under desiccating conditions [69]. More recently, polyphenolic compounds such as resveratrol and epigallocatechin gallate (EGCG) have been formulated into nanoemulsions or liposomes to enhance corneal penetration and therapeutic efficacy [70]. These natural compounds exhibit anti-inflammatory effects through inhibition of NF-κB and other key signaling pathways involved in DED pathogenesis [71,72].

### 4.3. Oral Supplementation

Oral supplementation constitutes another essential pillar of DED management, especially in chronic or refractory cases. Polyunsaturated fatty acids (PUFAs), in particular omega-3 fatty acids such as eicosapentaenoic acid (EPA) and docosahexaenoic acid (DHA), have demonstrated significant anti-inflammatory effects by modulating eicosanoid synthesis and reducing the expression of proinflammatory cytokines such as IL-1β and TNF-α [73]. Clinical trials have reported improvements in both subjective symptoms and objective parameters (e.g., Schirmer test, TBUT) following oral omega-3 supplementation [74]. Gamma-linolenic acid (GLA), an omega-6 fatty acid, also contributes to the biosynthesis of anti-inflammatory prostaglandins and is often combined with omega-3s in commercial preparations [75]. In addition, antioxidant micronutrients such as vitamins C and E, selenium, and zinc support ocular surface health by counteracting oxidative stress and contributing to epithelial regeneration [76]. The efficacy of these oral interventions appears to be particularly relevant in patients with Meibomian gland dysfunction (MGD) or systemic inflammatory comorbidities such as rosacea and Sjögren’s syndrome [77].

### 4.4. Anti-Inflammatory Pharmacotherapy

In moderate-to-severe DED, anti-inflammatory pharmacotherapy is essential to interrupt the vicious cycle of inflammation and epithelial damage. Topical corticosteroids offer rapid symptomatic relief and are effective in suppressing acute inflammation; however, their long-term use is limited by potential side effects such as elevated intraocular pressure and cataract formation [78]. Therefore, short pulses of low-potency corticosteroids (e.g., loteprednol) are preferred in flare-ups or during initiation of other therapies. Immunomodulatory agents, particularly cyclosporine A and lifitegrast, are used chronically to control T-cell–mediated inflammation and promote tear production. Cyclosporine A acts by inhibiting calcineurin signaling and IL-2 transcription, thereby reducing conjunctival inflammation and increasing goblet cell density [79]. Lifitegrast, an LFA-1 antagonist, blocks T-cell adhesion and cytokine release by disrupting the LFA-1/ICAM-1 interaction [80]. Both agents have been shown to improve ocular surface staining and patient-reported outcomes with continuous use over several months [80]. Tacrolimus and other immunosuppressants may be considered in refractory cases or in DED associated with autoimmune diseases [81].

Then, current treatments for DED mainly include lubricating eye drops and anti-inflammatory agents. However, these therapies have significant limitations, such as the need for frequent administration and the poor bioavailability of drugs on the ocular surface, which expose patients to discomfort and non-resolutive outcomes. These challenges are driving research toward alternative strategies for topical ophthalmic drug delivery.

## 5. Polyphenols and Nanotechnology: Innovative Strategies for Topical Ophthalmic Solutions in DED

**Figure 3:**
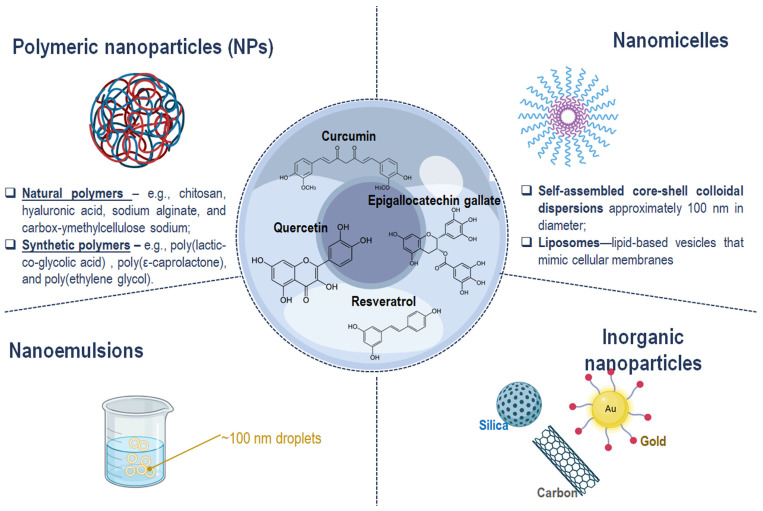
Schematic representation of nanotechnology-based strategies to enhance the therapeutic efficacy and tolerability of polyphenols in ocular treatment. This image was created with BioRender.com.

Given the multifactorial pathogenesis of DED, which involves inflammation, oxidative stress, ocular surface damage, and corneal nerve irritation, therapeutic agents must address these diverse mechanisms. In this context polyphenols have emerged as suitable candidates for prevention and treatment of chronic diseases whose pathogenesis is strictly due to oxidative stress-induced inflammation. Indeed, their antioxidant, anti-inflammatory, neuroprotective, and anti-angiogenic effects are key aspects influencing the potentiality in DED [82]. Polyphenolic compounds, such as epigallocatechin gallate (EGCG), resveratrol, quercetin, and curcumin, have attracted significant interest due to their potent antioxidant, anti-inflammatory, antimicrobial, antiviral, anti-aging, anticancer, and neuroprotective activities [83]. Their natural origin is often associated with improved safety profiles compared to synthetic drugs [84]. Consequently, polyphenols are increasingly regarded as valuable adjuvants or complementary agents in DED management [82]. However, the clinical translation of polyphenols is impeded by inherent limitations including poor aqueous solubility, chemical instability, rapid systemic clearance, and limited ocular bioavailability [85,86]. Moreover, the rapid washout of conventional topical eye drops from the ocular surface further diminishes therapeutic efficacy [87]. Nanoencapsulation strategies have thus been employed to enhance ocular retention, improve chemical stability, and increase bioavailability, thereby potentiating the clinical effectiveness of polyphenols in DED treatment (Figure 3) [87]. In addition to these well-recognized limitations, oral administration of polyphenols faces significant pharmacokinetic barriers related to gastrointestinal degradation, pH sensitivity, and interaction with gastric fluids that can lead to chemical transformation before absorption. The specific sites of intestinal uptake—mainly in the small intestine via passive diffusion and active transport mechanisms—further influence their systemic availability. Importantly, gut microbiota plays a key role in metabolizing polyphenols into bioactive or inactive metabolites, introducing marked interindividual variability that complicates prediction of systemic and ocular exposure. Such variability implies that only a minimal fraction of orally ingested polyphenols may realistically reach ocular tissues, which provides a strong rationale for advancing their development as topical nanoformulations designed for targeted ocular delivery [85]. Nanoformulation techniques improve polyphenol solubility and protect against enzymatic and oxidative degradation, enabling sustained drug release and reduced dosing frequency, which are critical for chronic disease management and patient adherence [7,88]. Additionally, nanoparticulate carriers facilitate transcorneal penetration, ensuring delivery of therapeutic concentrations to target ocular tissues [88]. Translational research has advanced several polyphenol nanoformulations from in vitro and animal studies to early-phase clinical trials. For instance, encapsulated EGCG in polymeric nanoparticles has demonstrated enhanced ocular bioavailability and significant mitigation of corneal inflammation and oxidative damage in preclinical DED models, supporting its clinical evaluation [89]. Similarly, curcumin-loaded nanoparticles have exhibited promising anti-inflammatory effects and promotion of corneal epithelial repair [90]. Complementary nanocarriers like nanomicelles, which are self-assembled core–shell colloidal dispersions approximately 100 nm in diameter, and liposomes—lipid-based vesicles that mimic cellular membranes—offer enhanced stability and permeability for ophthalmic applications [91]. Emerging nanomaterials, including nanoemulsions (~100 nm droplets), and inorganic nanoparticles composed of silica, gold, or carbon, have been tailored for targeted delivery to both anterior and posterior segments of the eye [92]. Table 1 summarizes how each nanocarrier addresses the challenges of solubility, stability, and bioavailability, along with their potential for clinical translation. However, it should be noted that polyphenols also possess prooxidant activity under certain conditions, such as high concentrations or in the presence of transition metal ions, which could potentially induce oxidative damage to ocular tissues. This dual nature underscores the importance of careful dose optimization and targeted delivery to maximize therapeutic benefits while minimizing potential adverse effects. Then, despite encouraging preclinical outcomes, the translation of these nanocarriers into clinical practice remains limited. A deeper understanding of how these experimental findings translate into therapeutic benefit for patients with DED—particularly in comparison with currently available therapies such as cyclosporine, lifitegrast, or corticosteroid-based formulations—is still needed. Considerations regarding long-term ocular safety, potential cumulative toxicity, and the maintenance of tear film integrity under chronic exposure are essential for their clinical acceptance. Moreover, large-scale manufacturing, batch-to-batch reproducibility, and compliance with Good Manufacturing Practices (GMPs) are critical for scalability and regulatory approval. Regulatory frameworks for nanomedicines are still evolving, and establishing standardized criteria for characterization, stability, and safety evaluation will be decisive for clinical translation [91,92].

In this section we critically evaluate preclinical evidence regarding specific polyphenol-nanocarrier combinations.

### 5.1. Curcumin

Curcumin (1,7-bis-(4-hydroxy-3-methoxyphenyl)-1,6-heptadiene-3,5-dione) is the principal polyphenolic compound isolated from Curcuma longa L. It is extensively utilized in the production of cosmetics, functional foods, and dietary supplements. Traditionally, curcumin has been employed in Chinese medicine for the management of diverse pathological conditions, primarily due to its potent anti-inflammatory, antioxidant, and antibacterial properties [93,94]. Importantly, curcumin has demonstrated therapeutic efficacy in multiple ophthalmic disorders affecting the anterior segment, including age-related macular degeneration, cataract, glaucoma, pterygium, and anterior uveitis, through modulation of various molecular pathways [95]. Mechanistically, curcumin exerts effects on mitochondrial oxidative stress, modulates inflammatory responses via peroxisome proliferator-activated receptor gamma (PPAR-γ)-dependent pathways, and downregulates pro-inflammatory enzymes such as cyclooxygenase-2 (COX-2) and inducible nitric oxide synthase (iNOS) [96]. Furthermore, it interferes with the JAK2-STAT3 signaling pathway, exhibiting anti-astrogliosis activity, and inhibits angiogenesis by targeting vascular endothelial growth factor (VEGF)/VEGF receptor (VEGFR) and K-ras pathways [97]. Additionally, curcumin suppresses the nuclear factor kappa B (NF-κB) transcription factor, induces the nuclear factor erythroid 2–related factor 2 (Nrf2) antioxidant pathway, and promotes expression of the tumor suppressor p53, which elevates pro-apoptotic Bax and cytochrome c levels [98]. Notably, curcumin undergoes autoxidation and degradation at physiological pH, and its early oxidative products influence the activity of topoisomerases, which are targets of various chemotherapeutic agents [99]. The anti-inflammatory activity of curcumin is particularly emphasized in ocular research. For instance, curcumin inhibits ovalbumin-induced proinflammatory cytokine expression in murine conjunctiva and mitigates hyperosmotic stress-induced upregulation of interleukin-1β (IL-1β) in corneal epithelial cells through the p38 mitogen-activated protein kinase (MAPK)/NF-κB pathway [100]. Given that proinflammatory cytokines such as IL-1β, IL-6, IL-8, IL-10, interferon-γ (IFN-γ), and tumor necrosis factor-α (TNF-α) are elevated in dry eye patients under hyperosmotic conditions, curcumin emerges as a promising alternative therapeutic agent for DED [101]. Despite these pharmacological potentials, curcumin’s clinical utility is limited by poor aqueous solubility (~11 ng/mL), chemical instability, and consequently low bioavailability—even at high oral doses up to 12 g/day. Oral administration results in peak serum concentrations below 50 ng/mL due to limited intestinal absorption and binding to enterocytes, which alter curcumin’s structure [102]. This necessitates repeated high dosing, often associated with gastrointestinal adverse effects. Therefore, topical ocular administration is favored for treating ophthalmic conditions, as it permits direct localization to ocular tissues, minimizing systemic exposure and related side effects [103]. To address the challenges of curcumin’s solubility and bioavailability, various nanotechnology-based approaches have been developed [104]. The first FDA-approved nanocarriers for curcumin were liposomes—spherical bilayer vesicles with favorable biodegradability, biocompatibility, and non-immunogenicity. These liposomes are typically composed of phosphatidylcholine, cholesterol, and lipid-conjugated hydrophilic polymers [105]. Nevertheless, liposomes suffer from chemical instability that may cause premature drug leakage [106]. To overcome these limitations, polymeric micelles and nanoparticle systems have been designed. Micelles consist of amphiphilic components such as hydrophilic poly(ethylene glycol) (PEG), chitosan, and polyvinyl pyrrolidone (PVP), paired with hydrophobic cores formed by poly(lactic acid) (PLA), poly(lactic-co-glycolic acid) (PLGA), distearoyl phosphoethanolamine (DSPE), dioleoyl phosphatidylethanolamine (DOPE), and vitamin E [107]. For instance, curcumin-loaded nanomicelles based on polyvinyl caprolactam-polyvinyl acetate-polyethylene glycol (PVCL-PVA-PEG) graft copolymers have demonstrated enhanced physicochemical stability, improved cellular uptake, superior corneal permeation, and increased anti-inflammatory efficacy both in vitro and in vivo compared to free curcumin solutions [107]. Furthermore, β-cyclodextrin-based nanoparticles modified with ethylenediamine (EDA) have been synthesized to augment thermodynamic stability, solubilization potential, and corneal permeability by modifying intermolecular hydrogen bonding within the nanoparticle cavity [108]. A critical consideration in ocular biomaterial design is prolonging the residence time on the ocular surface to reduce frequent administration. To this end, in situ gelling systems combining curcumin-loaded nanoparticles or mixed micelles have been developed. Thermosensitive polymers such as chitosan and pluronic facilitate a sol-to-gel phase transition at ocular surface temperature (~34 °C), providing sustained drug release and protection from degradation [109]. PLGA has been widely employed for its sustained-release properties and excellent biocompatibility, while chitosan offers mucoadhesive features through electrostatic and hydrogen bonding interactions with mucin’s sialic acid residues [110]. In this context, Cheng et al. developed a dual-drug delivery system comprising curcumin-loaded PLGA nanoparticles embedded within a chitosan-gelatin hydrogel, achieving prolonged therapeutic activity on the ocular surface [110]. Table 2 summarizes the cited examples.

### 5.2. Epigallocatechin Gallate (EGCG)

Epigallocatechin gallate (EGCG), chemically known as (−)-cis-2-(3,4,5-trihydroxyphenyl)-3,4-dihydro-1(2H)-benzopyran-3,5,7-triol-3-gallate, represents the most abundant and bioactive catechin in green tea. It has been extensively studied for its broad spectrum of pharmacological activities, including antibacterial, anticancer, anti-collagenase, antifibrotic, anti-inflammatory, and antioxidant properties, which are beneficial in modulating various physiological and pathological human processes [111]. In the context of ocular diseases, EGCG has shown promising effects in modulating inflammation and oxidative stress, key mechanisms underlying DED and related ocular surface disorders. Studies in human corneal epithelial cells demonstrated that EGCG reduces the release of proinflammatory cytokines/chemokines and decreases intracellular reactive oxygen species (ROS) levels in a dose-dependent manner [112]. Furthermore, EGCG inhibited the phosphorylation of MAPKs, including p38 and JNK, as well as the activation of transcription factors NF-κB and AP-1. These findings confirm the compound’s potential therapeutic efficacy in attenuating inflammatory and oxidative damage associated with DED and other ocular inflammatory conditions [113]. Despite its biological potency, the clinical application of EGCG is hindered by its poor aqueous solubility and low systemic bioavailability, characterized by rapid serum degradation and a short half-life [114]. To address these limitations, Shim et al. developed a formulation strategy based on polyethylene glycol (PEG)-mediated complexation. By combining EGCG with PEG and employing lyophilization to induce hydrogen bond formation, nanoscale PEG/EGCG complexes were synthesized, showing a 100-fold increase in solubility (up to 50 mg/mL) compared to the pure compound [115]. In a murine model of dry eye, these nanocomplexes significantly reduced inflammatory markers, including TNF-α, ICAM-1, VCAM-1, MMP-2, MMP-9, IL-17, IL-1β, and IL-6, thereby underscoring their therapeutic utility in DED [115]. Further advancements in ocular delivery of EGCG involved the development of gelatin-based nanoparticles, a strategy leveraging gelatin’s intrinsic biocompatibility and its structural similarity to corneal collagen [116]. To enhance mucoadhesive properties, hyaluronic acid was conjugated to the nanoparticle surface, generating gelatin–EGCG–hyaluronic acid (GEH) nanoparticles. In vitro, these particles exhibited high biocompatibility with human corneal epithelial cells (HCECs), while in vivo studies using a rabbit DED model confirmed a significant downregulation of proinflammatory genes IL1B and IL6 without detectable tissue inflammation or structural disruption [116]. To further improve ocular surface residence time and sustained EGCG delivery, a thermosensitive in situ gelling system was formulated. Luo et al. synthesized a gelatin-graft-poly(N-isopropylacrylamide) (GN) copolymer encapsulating EGCG, enabling sol–gel phase transition at physiological temperature [117]. In rabbit models, this system achieved therapeutic EGCG concentrations for up to three days post-application, reducing tear evaporation and preserving mucin-secreting goblet cells [117]. More recently, a long-acting mucoadhesive thermo-gel formulation for EGCG delivery was designed using gelatin as a degradable matrix, poly(N-isopropylacrylamide) as a thermo-responsive modulator, and lectin as a mucus-binding agent [118]. Topical application of this formulation in a rabbit model of DED significantly mitigated oxidative stress, inflammation, and apoptosis over a 14-day period, while promoting regeneration of the corneal epithelium [118]. These findings reinforce the value of EGCG as a viable candidate for polyphenol-based drug delivery systems aimed at the treatment of DED. Table 3 recaps the cited examples.

### 5.3. Resveratrol

Resveratrol (RSV), or 3,4′,5-trihydroxystilbene, is a naturally occurring phenolic stilbenoid identified in over 70 plant species, including berries, peanuts, mulberries, rhubarb, and particularly grapes. As such, wine constitutes a well-recognized dietary source of RSV, especially within the context of the Mediterranean diet [119]. RSV exists in two isomeric forms, cis- and trans-resveratrol, the latter being the most abundant and biologically active form found in food sources [120]. Numerous studies have demonstrated the wide-ranging biological properties of RSV, including antioxidant, anti-inflammatory, antibacterial, antibiofilm, cardioprotective, anticancer, and anti-aging effects [121,122]. The anti-inflammatory activity of RSV has been extensively studied due to its clinical relevance in various chronic diseases. RSV modulates inflammatory responses through the regulation of molecular mediators such as interleukins, and by interfering with key signaling pathways including the arachidonic acid (AA) cascade, nuclear factor-kappa B (NF-κB), mitogen-activated protein kinase (MAPK), and activator protein-1 (AP-1) [123,124]. Additionally, the phenolic ring structure of RSV allows for interaction with the active site of tyrosyl-tRNA synthetase, influencing the downstream activation of stress-related signaling cascades involved in tissue damage and inflammatory pathologies [125]. Furthermore, RSV is known to enhance the activity of sirtuin 1 (SIRT1), which in turn regulates p53 acetylation and DNA repair enzymes, thereby supporting the treatment of age-associated diseases [126]. RSV also directly interacts with peroxisome proliferator-activated receptors (PPARγ and PPARα), which are involved in enhancing insulin sensitivity and mediating anti-inflammatory effects in hepatic, adipose, and vascular tissues [126,127]. The therapeutic potential of RSV has also been explored in ophthalmology, notably for age-related macular degeneration and dry eye disease (DED) [128]. In DED, RSV demonstrated significant anti-inflammatory activity by reducing IL-1 levels and CD4^+^ T cell infiltration in tear fluid of murine models [129]. As an antioxidant, RSV was shown to restore mitochondrial dysfunction in human corneal epithelial (HCE-2) cells exposed to hyperosmotic stress. In this condition, SIRT1 expression was downregulated, leading to increased apoptosis and ROS production, along with reduced levels of antioxidant enzymes SOD2 and GPx. Treatment with RSV reversed these effects, reactivating SIRT1 expression, reducing oxidative stress, and improving both tear production and goblet cell density in vivo [129]. Despite these beneficial effects, RSV suffers from poor aqueous solubility, rapid systemic absorption, and low bioavailability, limiting its clinical translation [88,130]. To overcome these challenges, various nanotechnology-based approaches have been investigated. Among these, solid lipid nanoparticles (SLNs) and nanostructured lipid carriers (NLCs) have been extensively utilized to improve RSV stability and delivery [131]. For example, a resveratrol nanosuspension was prepared and characterized using dynamic light scattering, field emission scanning electron microscopy, and infrared spectroscopy. The RSV-NS exhibited an average particle size of 304.0 ± 81.21 nm, with a PDI of 0.225 ± 0.036, showing spherical-like morphology and a uniform particle distribution. Biological evaluation on human microvascular retinal endothelial cells (HMRECs) demonstrated that RSV-NS at concentrations below 18.75 µM was non-cytotoxic, while at 37.5 µM it significantly reduced cell proliferation and migration compared to the unstimulated control. These findings suggest that RSV-NS not only provides a safe nanoscale formulation but also exerts functional regulation of endothelial cell behavior [132]. Moreover, a polymeric system composed of mucoadhesive lecithin/chitosan nanoparticles (RMLCNs) was developed to transport RSV to the anterior segment of the eye. Using a Quality by Design approach, nanoparticles with an average diameter of 163.3 nm were obtained, exhibiting strong mucoadhesive properties due to their high cationic surface charge. The formulation achieved an encapsulation efficiency of 97.03% and a sustained release profile of 96.87% over 8 h. Pharmacokinetic parameters such as the area under the curve (AUC_0_–_6_h) and mean residence time (MRT) were significantly improved—6.44-fold and 2.46-fold, respectively—compared to RSV in solution. These features underscore the formulation’s potential for scale-up and clinical application in ocular therapy [133]. Recently, micelle-based systems have emerged as a promising platform for ophthalmic drug delivery, inspired by commercially available ocular formulations. A Soluplus-based micellar system (Sol-RSV), composed of polyvinyl caprolactam-polyvinyl acetate-polyethylene glycol graft copolymer, was synthesized for the ophthalmic delivery of RSV. Sol-RSV demonstrated excellent chemical and storage stability, and no in vitro or in vivo toxicity. Compared to free RSV, this formulation exhibited enhanced passive permeation, cellular uptake, and corneal penetration in vivo. Moreover, Sol-RSV promoted corneal wound healing and modulated inflammatory and oxidative stress-related markers, thereby highlighting its therapeutic relevance in DED [134]. Also trimethylated chitosan-coated flexible liposomes (TMC-Lipo) could serve as an effective resveratrol delivery platform. By combining the antioxidant and anti-inflammatory properties of resveratrol with the mucoadhesive and barrier-penetrating features of TMC-Lipo, this system could counteract key pathogenic mechanisms of DED. Specifically, resveratrol-loaded TMC-Lipo may protect corneal and conjunctival epithelial cells from oxidative stress, restore mitochondrial function, and suppress inflammatory mediators such as IL-1β, TNF-α, and MMP-9, which are upregulated in DED. Moreover, the prolonged ocular surface residence time afforded by TMC enhances bioavailability, reducing dosing frequency and improving therapeutic outcomes in patients with chronic DED [135]. In Table 4 were summarized the described examples.

### 5.4. Quercetin

Quercetin (QUE), chemically defined as 3,3′,4′,5,7-pentahydroxyflavone, is a flavonol widely distributed across a variety of fruits and vegetables, with particularly high concentrations in apples, cherries, onions, and asparagus [136]. Both QUE and its glycoside derivatives have been extensively studied for their broad spectrum of biological activities, including antioxidant, anti-inflammatory, anti-fibrotic, immunomodulatory, and anti-cancer properties [137]. Additionally, QUE has shown potential in the treatment of bacterial and viral infections, as well as in inhibiting biofilm formation [138]. The antioxidant and anti-inflammatory effects of QUE have also been exploited in ophthalmological research, with applications reported in keratoconus, Graves’ ophthalmopathy, conjunctivitis, cataract, retinopathy, and particularly in dry eye disease (DED) [139]. It is well established that QUE modulates intracellular reactive oxygen species (ROS) levels under conditions of hypoxia, injury, or mitochondrial dysfunction, thereby attenuating the activation of the nuclear factor-kappa B (NF-κB) pathway [140,141]. This regulatory mechanism helps to mitigate chronic inflammation and pathological neovascularization. Furthermore, QUE has been shown to downregulate interleukin (IL)-6 and IL-10 production in a dose-dependent manner. Since reduced tear secretion compromises ocular surface immunity, the potential of QUE to counteract inflammation in DED has been investigated. In vivo studies demonstrated that topical administration of QUE exerted immunomodulatory effects in murine models of DED [142]. Despite these promising findings, the clinical application of QUE in ophthalmic formulations is hindered by its poor aqueous solubility and limited chemical stability [143]. To address these challenges, various nanotechnological strategies have been developed to improve its solubility, bioavailability, and pharmacological efficacy [143]. Lipid-based nanocarriers have emerged as promising platforms for the ocular delivery of quercetin in DED, owing to their ability to protect lipophilic compounds, enhance physicochemical stability, and enable controlled release. Among these, nanoemulsions and solid lipid nanoparticles have been extensively investigated as delivery vehicles. Formulated with biocompatible surfactants such as Tween and Span to stabilize oil-in-water interfaces, these carriers typically exhibit particle sizes ranging from 150 to 345 nm, as confirmed by dynamic light scattering and transmission electron microscopy. Ocular delivery of quercetin has been validated through confocal imaging and ex vivo porcine eye models, with SLNs showing superior performance. Specifically, quercetin-loaded SLNs achieved the highest corneal flux (158 μg cm^−2^ after 24 h), while also demonstrating the lowest cytotoxicity on corneal and retinal ganglion cells, with IC_50_ values of 268.85 and 211.3 μg mL^−1^, respectively. Importantly, SLNs were able to protect ocular surface and neuronal cells from H_2_O_2_-induced oxidative injury, underscoring their therapeutic potential in mitigating oxidative stress, a key driver of DED pathophysiology [144]. Cyclodextrins (CDs), due to their cyclic oligosaccharide structure, have been employed as versatile excipients in drug formulation to enhance the solubility of lipophilic compounds like QUE. In particular, Lan et al. developed a novel ophthalmic delivery system incorporating hydroxypropyl-β-cyclodextrin (HP-β-CD) complexed with QUE and coated with a chitosan-N-acetyl-L-cysteine (CS-NAC) conjugate. This formulation improved both corneal penetration and ocular tissue distribution of QUE. The thiolated chitosan derivative (CS-NAC) provided superior mucoadhesive properties compared to unmodified chitosan, thereby prolonging retention time on the ocular surface [145]. Furthermore, binary and tertiary cyclodextrin complexes were formulated combining QUE with resveratrol (RSV), with or without the addition of hyaluronic acid (HA), to enhance solubility and chemical stability [146]. In vitro studies using human corneal and conjunctival cell lines revealed that both QUE and RSV, at their highest tested concentrations, scavenged over 95% of ROS. However, the ternary complexes exhibited superior stabilizing effects compared to the binary systems [146]. Polymeric nanoparticles represent another promising approach for quercetin delivery in Dry Eye Disease (DED). Quercetin has been successfully encapsulated into poly(lactic-co-glycolic acid) (PLGA) nanoparticles using a solvent displacement method, yielding formulations with favorable physicochemical stability and controlled degradation kinetics. In vitro assays demonstrated that quercetin nanoparticles exert potent antioxidant activity, which was further enhanced when combined with epigallocatechin gallate (EGCG), indicating an additive effect on free radical scavenging. Importantly, these nanoparticles showed superior intracellular inhibition of reactive oxygen species (ROS) in comparison to N-acetyl cysteine, underscoring their strong cytoprotective potential. To optimize ocular administration, a thermosensitive gel incorporating quercetin nanoparticles and EGCG was developed, exhibiting an appropriate gelation temperature and transition time for topical application. This system ensured sustained release, prolonged residence on the ocular surface, and enhanced antioxidant protection of human corneal epithelial cells [147]. Table 5 recaps the cited examples.

## 6. Challenges and Future Perspectives

Despite the promising preclinical results, the translation of polyphenol-loaded nanocarriers into effective clinical therapies for DED faces several challenges. A major limitation lies in the intrinsic physicochemical variability of polyphenols, which often exhibit poor solubility, instability under physiological conditions, and rapid metabolism, leading to inconsistent therapeutic outcomes [148,149]. Moreover, the reproducibility and scalability of nanocarrier fabrication processes—particularly for lipid- and polymer-based systems—remain critical barriers to clinical translation. Variability in particle size, encapsulation efficiency, and drug release kinetics can affect bioavailability and ocular retention, complicating regulatory approval and large-scale manufacturing [150]. Another significant challenge is the lack of standardized in vitro and in vivo models that accurately replicate the complex pathophysiology of human DED, including tear film dynamics, inflammation, and oxidative stress. Current animal models often fail to predict clinical performance, emphasizing the need for more physiologically relevant experimental systems and advanced imaging or biomarker-based tools to monitor therapeutic efficacy [151]. Furthermore, the ocular surface presents formidable biological barriers—such as tear turnover, mucin layer interactions, and corneal permeability—that limit sustained drug residence and penetration, even with nanocarrier assistance [152]. From a clinical perspective, the long-term safety of chronic topical exposure to nanomaterials must be thoroughly evaluated, as potential issues such as ocular irritation, immune activation, or nanoparticle accumulation may emerge with prolonged use [153]. Regulatory pathways for nanomedicines are also still evolving, and harmonization of safety and quality assessment guidelines is essential to accelerate clinical translation [154]. Future research should prioritize the development of personalized nanotherapeutic strategies, integrating patient-specific factors such as tear composition, inflammatory profile, and oxidative status to optimize treatment response. Combining polyphenols with other synergistic agents or hybrid nanocarrier platforms (e.g., lipid–polymer conjugates or stimuli-responsive systems) may further enhance drug stability and bioavailability. Advances in microfluidic fabrication, 3D ocular models, and omics-based approaches will improve formulation precision, mechanistic understanding, and predictive capacity. Finally, the interdisciplinary collaboration among formulation scientists, ophthalmologists, and regulatory authorities is necessary to ensure safe, reproducible, and effective nanopolyphenol therapies for DED management.

## 7. Conclusions

Polyphenols represent a promising class of bioactive molecules for the treatment of DED, owing to their wide-ranging pharmacological actions, including antioxidant, anti-inflammatory, anti-apoptotic, and immunomodulatory properties. However, the translation of these natural compounds into clinical ophthalmic therapies is hindered by significant biopharmaceutical limitations, such as poor aqueous solubility, low chemical and enzymatic stability, rapid metabolism, and limited transcorneal permeability. This review highlighted four representative polyphenols—epigallocatechin gallate (EGCG), curcumin, resveratrol, and quercetin—that have been encapsulated in a variety of nanocarriers to improve their therapeutic performance in preclinical models of DED. The encapsulation of EGCG in chitosan-based or gelatin-based nanoparticles has demonstrated notable anti-inflammatory and antioxidative effects, reducing corneal damage and modulating cytokine expression. Curcumin, when formulated in micelles or lipid nanocarriers, showed improved chemical stability and targeted delivery, leading to enhanced bioactivity in vitro and in vivo. Resveratrol-loaded systems, including micelles and mucoadhesive nanoparticles, exhibited restored tear film homeostasis and promoted mitochondrial function via SIRT1 modulation. Similarly, quercetin was successfully incorporated into lipidic nanocarriers and cyclodextrin-based systems, improving its ocular retention and reducing oxidative stress, inflammatory cytokines, and CD4^+^ T cell infiltration in DED models. The promising results from these nanoformulations highlight the ability of nanotechnology to not only overcome the intrinsic limitations of polyphenols but also to enhance their tissue-specific targeting, residence time on the ocular surface, and biological efficacy. Nanocarriers such as solid lipid nanoparticles (SLNs), nanostructured lipid carriers (NLCs), micelles, and cyclodextrin conjugates offer unique advantages, including controlled drug release, increased mucoadhesion, and enhanced patient compliance. Moreover, the ability to modulate critical molecular pathways involved in DED—such as NF-κB, MAPK, SIRT1, VDR, and AP-1—further supports the therapeutic relevance of these strategies. In the next future, nanotechnology devices applied to DED treatment would benefit from a more systematic and comparative evaluation of different polyphenol-loaded nanocarriers, focusing on optimizing formulation parameters, dosing regimens, and routes of administration. The potential synergistic effects of combining multiple polyphenols—such as the resveratrol-quercetin association—deserve deeper investigation, particularly with the development of co-delivery systems that can release both agents in a temporally and spatially controlled manner. Moreover, future studies must address regulatory challenges, conduct long-term safety assessments, and establish scalable manufacturing protocols. Overall, with further advancements and interdisciplinary collaboration, polyphenol-loaded nanodevices have the potential to significantly enhance the efficacy, safety, and personalization of DED management, ultimately improving patients’ quality of life.

## Figures and Tables

**Table 1:** Potential of nanocarriers for clinical translation.

Nanocarrier	Solubility Enhancement	Stability Improvement	Bioavailability Improvement	Potential for Clinical Translation
**Polymeric nanoparticles**	Encapsulation of hydrophobic polyphenols improves aqueous solubility by entrapping them in a hydrophilic matrix.	Protects polyphenols from enzymatic and oxidative degradation through polymer encapsulation.	Sustained drug release and prolonged ocular residence time; improved transcorneal penetration.	Advanced to early-phase clinical trials for DED; promising preclinical results support translation.
**Nanomicelles**	Self-assembled core–shell structure increases solubility of hydrophobic drugs by providing a hydrophilic shell.	Protects labile compounds by sequestering them in the micelle core, shielding from environmental factors.	Enhances ocular penetration due to nanosize and favorable surface properties; prolonged residence.	Emerging approach with promising stability and solubility profiles; needs more clinical studies.
**Liposomes**	Encapsulate both hydrophobic and hydrophilic drugs, improving solubility.	Lipid bilayer protects encapsulated drugs from hydrolysis and enzymatic degradation.	Improves drug residence time, bioavailability, and corneal penetration.	Well-established drug delivery system with some ophthalmic applications already in clinical use; regulatory pathways more defined.
**Nanoemulsions**	Disperse hydrophobic compounds in nanosized droplets, enhancing solubility in aqueous media.	Physical stability through emulsification; protects against chemical degradation.	Small droplet size improves penetration and bioavailability; sustained release potential.	Good potential for ophthalmic application; requires formulation optimization for clinical translation.
**Inorganic nanoparticles (silica, gold, carbon)**	Can be engineered to incorporate hydrophobic polyphenols or coat with hydrophilic layers for solubility improvement.	High chemical and physical stability; resistant to enzymatic degradation.	Tailorable surface properties enhance ocular penetration; possibility of targeted delivery.	Innovative but early stage; regulatory challenges remain due to safety concerns and lack of large-scale studies.

**Table 2:** Curcumin based ocular drug delivery systems.

Delivery System	Key Characteristics	Reference
**PVCL-PVA-PEG nanomicelles**	Enhanced physicochemical stability, improved cellular uptake, superior corneal permeation, increased anti-inflammatory efficacy (in vitro & in vivo)	[107]
**β-cyclodextrin nanoparticles modified with ethylenediamine (EDA)**	Improved thermodynamic stability, solubilization potential, and corneal permeability via modified intermolecular H-bonding	[108]
**In situ gelling systems with curcumin-loaded nanoparticles or mixed micelles (using chitosan, pluronic)**	Thermosensitive sol-to-gel transition at ocular temperature (~34 °C); sustained release; protection from degradation	[109]
**Curcumin-loaded PLGA nanoparticles in chitosan–gelatin hydrogel (dual system)**	Prolonged ocular surface retention, extended therapeutic activity	[110]

**Table 3:** Epigallocatechin gallate based ocular drug delivery systems.

Delivery System	Key Characteristics	Reference
**PEG/EGCG nanocomplexes (PEG-mediated complexation, lyophilized)**	100-fold solubility increase (up to 50 mg/mL); reduced inflammatory markers (TNF-α, ICAM-1, VCAM-1, MMP-2, MMP-9, IL-17, IL-1β, IL-6) in murine DED model	[115]
**Gelatin-based nanoparticles (GEH: gelatin–EGCG–hyaluronic acid)**	Biocompatible with HCECs; hyaluronic acid improved mucoadhesion; in vivo downregulated IL1B and IL6 without tissue damage in rabbit DED model	[116]
**Gelatin-graft-poly(N-isopropylacrylamide) (GN) copolymer (in situ gelling system)**	Thermosensitive sol–gel transition at physiological temperature; sustained EGCG release for 3 days; reduced tear evaporation; preserved goblet cells in rabbit model	[117]
**Long-acting mucoadhesive thermo-gel (gelatin + PNIPAM + lectin)**	Prolonged ocular surface retention; mitigated oxidative stress, inflammation, and apoptosis; promoted corneal epithelium regeneration over 14 days in rabbit DED model	[118]

**Table 4:** Resveratrol based ocular drug delivery systems.

Delivery System	Key Characteristics	Reference
**Resveratrol nanosuspension (RSV-NS)**	Particle size: 304.0 ± 81.21 nm; PDI: 0.225 ± 0.036; spherical morphology, uniform distribution. Non-cytotoxic below 18.75 µM; reduced proliferation and migration of HMRECs at 37.5 µM	[132]
**Lecithin/chitosan nanoparticles (RMLCNs)**	Mucoadhesive polymeric system; particle size: 163.3 nm; cationic surface charge; encapsulation efficiency: 97.03%; sustained release: 96.87% over 8 h; AUC_0_–_6_h * ↑ 6.44-fold, MRT * ↑ 2.46-fold vs. free RSV	[133]
**Soluplus-based micelles (Sol-RSV)**	PVCL-PVA-PEG micellar system; excellent chemical/storage stability; safe in vitro & in vivo; enhanced permeation, cellular uptake, and corneal penetration; promoted corneal wound healing; modulated oxidative stress and inflammation markers	[134]
**Trimethylated chitosan-coated flexible liposomes (TMC-Lipo)**	Mucoadhesive and barrier-penetrating; improved ocular surface residence time; protected epithelial cells from oxidative stress; restored mitochondrial function; suppressed IL-1β, TNF-α, and MMP-9; improved bioavailability and therapeutic outcomes in DED	[135]

* ↑ indicates increased value.

**Table 5:** Quercetin based ocular drug delivery systems.

Delivery System	Key Characteristics	Reference
**Nanoemulsions (NEs) and Solid Lipid Nanoparticles (SLNs)**	Particle size: 150–345 nm; stabilized with Tween/Span; validated ocular delivery via confocal microscopy and ex vivo porcine eyes. Quercetin-SLNs: highest corneal flux (158 μg cm^−2^/24 h); lowest cytotoxicity (IC_50_: 268.85 μg/mL for corneal cells, 211.3 μg/mL for retinal ganglion cells); protected cells from H_2_O_2_-induced oxidative injury	[144]
**HP-β-CD/Quercetin complex coated with CS-NAC**	Improved corneal penetration and ocular tissue distribution; enhanced solubility; superior mucoadhesive properties of CS-NAC prolonged ocular surface retention compared to unmodified chitosan	[145]
**Binary and ternary cyclodextrin complexes (Quercetin + Resveratrol ± Hyaluronic Acid)**	Enhanced solubility and chemical stability; in vitro ROS scavenging > 95% in corneal and conjunctival cells; ternary complexes provided superior stabilization compared to binary complexes	[146]
**PLGA nanoparticles (Quercetin-NPs) ± EGCG in thermosensitive gel**	Stable nanoparticles with controlled degradation; strong antioxidant activity with additive effect of EGCG; superior intracellular ROS inhibition vs. N-acetyl cysteine; thermosensitive gel showed appropriate gelation and sustained release; prolonged ocular residence; enhanced antioxidant protection of corneal epithelial cells	[147]

## Data Availability

Not applicable.
